# Near-Infrared Spectroscopy Applied to the Detection of Multiple Adulterants in Roasted and Ground Arabica Coffee

**DOI:** 10.3390/foods11010061

**Published:** 2021-12-28

**Authors:** Cinthia de Carvalho Couto, Otniel Freitas-Silva, Edna Maria Morais Oliveira, Clara Sousa, Susana Casal

**Affiliations:** 1 Food and Nutrition Graduate Program, Federal University of State of Rio de Janeiro, Av. Pasteur 296, Rio de Janeiro 22290-240, Brazil; cinthiaccouto@gmail.com; 2 Embrapa Food Agroindustry, Av. das Américas 29501, Rio de Janeiro 23020-470, Brazil; otniel.freitas@embrapa.br (O.F.-S.); edna.oliveira@embrapa.br (E.M.M.O.); 3 CBQF—Centro de Biotecnologia e Química Fina, Laboratório Associado, Escola Superior de Biotecnologia, Universidade Católica Portuguesa, Rua Diogo Botelho 1327, 4169-005 Porto, Portugal; 4 LAQV/REQUIMTE, Laboratory of Bromatology and Hydrology, Faculty of Pharmacy, University of Porto, 4050-313 Porto, Portugal; sucasal@ff.up.pt

**Keywords:** coffee, adulteration, infrared spectroscopy, authenticity, chemometrics

## Abstract

Roasted coffee has been the target of increasingly complex adulterations. Sensitive, non-destructive, rapid and multicomponent techniques for their detection are sought after. This work proposes the detection of several common adulterants (corn, barley, soybean, rice, coffee husks and robusta coffee) in roasted ground arabica coffee (from different geographic regions), combining near-infrared (NIR) spectroscopy and chemometrics (Principal Component Analysis—PCA). Adulterated samples were composed of one to six adulterants, ranging from 0.25 to 80% (*w*/*w*). The results showed that NIR spectroscopy was able to discriminate pure arabica coffee samples from adulterated ones (for all the concentrations tested), including robusta coffees or coffee husks, and independently of being single or multiple adulterations. The identification of the adulterant in the sample was only feasible for single or double adulterations and in concentrations ≥10%. NIR spectroscopy also showed potential for the geographical discrimination of arabica coffees (South and Central America).

## 1. Introduction

Coffee is among the most consumed beverages worldwide [1], having enormous economic relevance, and has a continuously growing market, expanding to different applications, such as the cosmetic and pharmaceutical industries [2]. According to the International Coffee Organization (ICO), the global coffee output achieved near 172 million bags in 2020/21, represented by the main commercialized species, *Coffea arabica* (59%) and *Coffea canephora* (robusta) (41%). Brazil is the main coffee producer and exporter worldwide, with a total production estimated in the crop year 2020/2021 of 69 million bags (arabica and robusta), followed by Vietnam (mainly robusta) and Colombia (arabica), with 29 and 14.3 million bags, respectively [3,4].

Due to its commercial value, arabica coffee has been the target of countless and increasingly complex adulterations over the years [5], mainly through the addition of roasted barley, corn, rice and coffee husks [6,7]. Robusta coffee, due to its lower market and compositional similarity, is also commonly used for arabica coffee adulterations [1,7,8].

A plethora of studies have been developed to tentatively detect adulterations in roasted ground coffee employing physical, chemical, and biological techniques. Some include DNA-based approaches [9,10,11,12,13], chromatographic analysis [14,15], ultraviolet–visible spectrophotometry (UV–VIS) [16], digital image processing [17], capillary electrophoresis tandem mass spectrometry [18], electrospray ionization mass spectrometry [19], etc. However, these techniques require sophisticated and expensive instrumentation, as well as skilled personnel, are generally time-consuming, include chemical pre-treatments that make them destructive [20,21] and allow for the detection of only a few contaminants [22]. Microscopic inspection, one of the oldest approaches, is still commonly applied, including in official laboratories [23], despite its recognized incapacity to distinguish accurately multiple and complex contaminations, together with its inherent subjectivity, highly based on the analyst’s experience [7]. More expedite methods are deemed necessary to effectively support adulteration detection worldwide [24,25].

Some vibrational spectroscopic techniques, such as NIR spectroscopy and NMR, coupled with chemometrics have already proved to be reliable tools in the detection of particular coffee adulterations [7,19,24,26,27]. These techniques are well known for their high efficiency, fastness, reliability and easy use. They commonly do not demand sample pre-treatments nor reagents, showing to be green analytical tool alternatives [7,28,29]. NIR spectroscopy has been widely used to discriminate arabica and robusta species, in both green and roasted coffee [30], and even to correlate with sensorial attributes in roasted coffee [31]. The detection of different adulterants in coffee through NIR, such as corn, barley and coffee husks, has also been reported [24,25,29], but not yet extensively tested for the detection of mixtures, increasingly used as coffee adulterants [13,21,26]. In most published works, only one or two adulterants per sample have been tested, which does not represent the reality of the actual market. Therefore, models should be more representative, composed of a wider variety of coffee origins and adulterants simultaneously in the same sample. Additionally, the most likely types of combinations of the different varieties and mixtures must be considered [21,24]. Recently, advances in NMR have been made, demonstrating the versatility of this technique for the detection of multiple adulterants [32] but not, as far as the authors know, for NIR spectroscopy.

Considering the lack of information on some of the most recent materials used for coffee fraud, and the increased use of multiple adulterations, this work aimed to study the feasibility to detect multiple coffee adulterants in roasted and ground coffee, in different combinations, based on NIR spectral information.

## 2. Materials and Methods

### 2.1. Raw Material

Roasted coffee beans were kindly selected and provided by Nestlé roaster (Porto, Portugal). Sampling was representative of the main species commercialized, including different geographical origins as well as the main producers and exporters of coffee. Four arabica roasted samples were used: two from Brazil (both natural), and one each from Colombia and Honduras (both washed—“milds”). Two robusta roasted samples were used as adulterants, from Vietnam and Cameroon. All coffee beans were ground (Retsch, GM 200, Haan, Germany) and stored at room temperature under light and air protection until analysis using aluminum bags with one-way valves as usual in the coffee industry.

The remaining adulterants (corn, soybeans, rice seeds, barley and the dried residues from natural coffee processing, commonly known as coffee husks) were chosen considering the most recent trends in commercial roasted and ground coffee adulteration (Table 1) [6,11,27]. Two distinct batches of each adulterant were acquired (1 and 2), roasted to achieve a color similar to that of the coffees used (medium dark) in a laboratory oven (WTC Binder, Tuttlingen, Germany) (Table 1) and ground (Retsch, GM 200, Haan, Germany), except barley which was already acquired roasted and ground in the local market.

The blends (adulterated arabica coffee) were prepared with a single adulterant up to all the six adulterants together, in different mass percentages (0.25, 0.5, 1, 5, 10, 20, 40, 60 and 80%) and combinations. All the blends were prepared in triplicate. Briefly, the 0.25% and 0.5% adulterations were only prepared with single adulterants, while the 40, 60 and 80% adulterations were only prepared with robusta coffee as adulterant. The adulterations between 1 and 30% resulted either from individual adulterations or from combinations of two to six adulterants. The 2% frauds, for example, resulted from the blend of two adulterants at 1% and from combination of 4 adulterants at 0.5%. The 5%, similarly, was the result of individual adulterations at 5% or from combination of five adulterants at 1%. Only a single adulteration at 25% and 30% was prepared, resulting from using five and six adulterants at 5%, respectively. Single adulterations at 20% were only prepared with corn, coffee husks and robusta coffee, although 20% fraud could result from a combination of two (at 10%) or four (at 5%) adulterations. Globally, a total of 73 combinations were prepared, in triplicate, totaling 219 adulterated samples. For details, please see Tables S1 and S2 (Supplementary Materials).

### 2.2. Near-Infrared Spectroscopy

Near-infrared spectra of all the samples were acquired on a Fourier-transform near-infrared spectrometer (FTLA 2000, ABB, Québec, QC, Canada) equipped with an indium-gallium-arsenide (InGaAs) detector in diffuse reflectance mode. Each spectrum resulted from an average of 64 scans with a resolution of 8 cm^−1^ in the wavenumber interval of 4000–10,000 cm^−1^. Bomen-Grams software (version 7, ABB, Québec, QC, Canada) was used to control the equipment. A total of five spectra per sample were acquired for each sample triplicate (meaning a total of 15 spectra for each plain sample of coffee and adulterant plus all the 291 blends prepared). All the analysis took place within 6 months after roasting.

### 2.3. Data Analysis

Due to the large amount of spectral data, the 5 spectra of each sample were averaged before data analysis. The mean spectra were pre-processed with standard normal variate (SNV) and Savitzky-Golay filter (15 smoothing points, 2nd order polynomial and 1st derivative) [33] to remove baseline drifts and further mean centered. Other data pre-treatments were tested as: (I) different combinations of SNV and SavGol filter (SNV + mean center; SavGol + mean center); (II) different windows of the SavGol filter (9–15) and also the second derivative; (III) multiplicative scatter correction (MSC) and (IV) autoscale. It should be stressed that the best results were obtained with the above-mentioned pre-treatment. Spectra were further modelled by Principal component analysis (PCA) [34]. Outliers were verified by Q Residuals versus Hotelling T^2. The root mean square errors of calibration (RMSEC) and cross validation (RMSECV) of all the PCA models developed in the current study were presented in Table S3 (Supplementary Materials). All chemometric models were performed in Matlab version 9.5 Release 2018b (MathWorks) and PLS Toolbox version 8.7 (2019) for Matlab (Eigenvector Research, Manson, WA, USA).

## 3. Results and Discussion

### 3.1. Discrimination among Pure Samples and Adulterated Coffee

**Figure 1:**
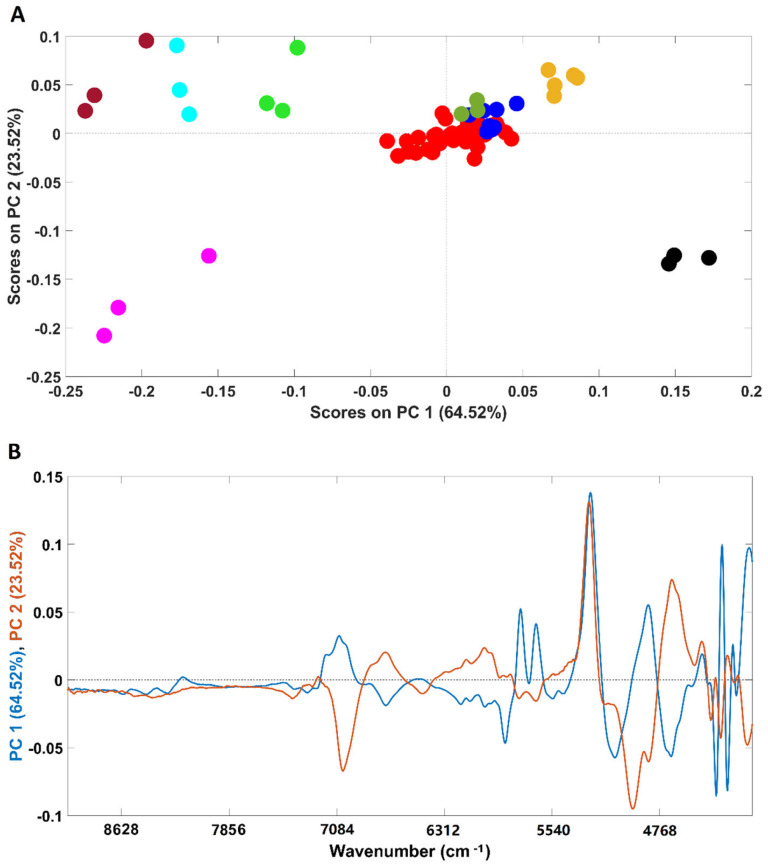
Scores plot of the PCA models developed with all the samples included in this study (**A**) and their corresponding loadings (**B**). Legend: ● arabica; ● robusta; ● adulterated samples with robusta; ● adulterated samples with rice/corn/soy/barley/coffee husks; ● soy; ● barley; ● rice; ● corn; ● coffee husks.

An exploratory PCA was performed to evaluate possible clusterization among all the analyzed samples (Figure 1A). The analysis was performed considering the whole spectral range (4000–10,000 cm^−1^). Spectra were pre-processed prior to the analysis (for details, please see the Materials and Methods section).

NIR spectroscopy was able to clearly discriminate the pure adulterants (rice, barley, soybean, corn and coffee husks) from samples containing coffee (robusta, arabica and arabica adulterated with robusta). It should be stressed that the first PC (PC1) mainly accounts for the discrimination between corn, rice, barley and soybean samples (negative part of PC1) from coffee husks (positive part of PC1). According to the loadings plot (Figure 1B), the wavenumber regions/bands that mostly account for such discrimination (higher-intensity bands) were: (I) the region between 5800 and 5650 cm^−1^ which are due to S-H and C-H bonds in first overtone; (II) peaks around 4360 and 4270 associated with the C-H plus C=C combination and at 4324 cm^−1^, a vibration attributed to lipids. It should be noted that, despite being high in intensity, the bands around 5200 and 7000 cm^−1^ are associated with the O-H combination and the first O-H overtones regions, respectively, due to the presence of water bands [35] and should not be taken into consideration for sample discrimination. Additionally, corn, rice and barley samples were closer in the scores map of PCA (Figure 1A) denoting a higher similarity when compared with soybean ones, discriminated across PC2. The spectral bands that seem to account for the discrimination are located at 4960 and 4671 cm^−1^, corresponding to a spectral range dominated by C-H plus C=C vibrations, and at 4324 cm^−1^, frequently attributed to lipid vibrations. Regarding the samples containing only coffee, they are closer in the scores map, with the four plain “arabica” samples being the most dissimilar ones. It is interesting to note that plain “robusta” and “arabica” samples adulterated with “robusta” cluster together, with the remaining adulterated samples lying in the top of the cluster closer to the “arabica” samples. The results obtained with the PCA demonstrate the high potential of this technique to discriminate among pure and adulterated coffee samples. Previous studies already demonstrated the suitability of NIR spectroscopy to discriminate among “arabica” and “robusta” varieties, which are in accordance with the results herein obtained [8,29,30].

**Figure 2:**
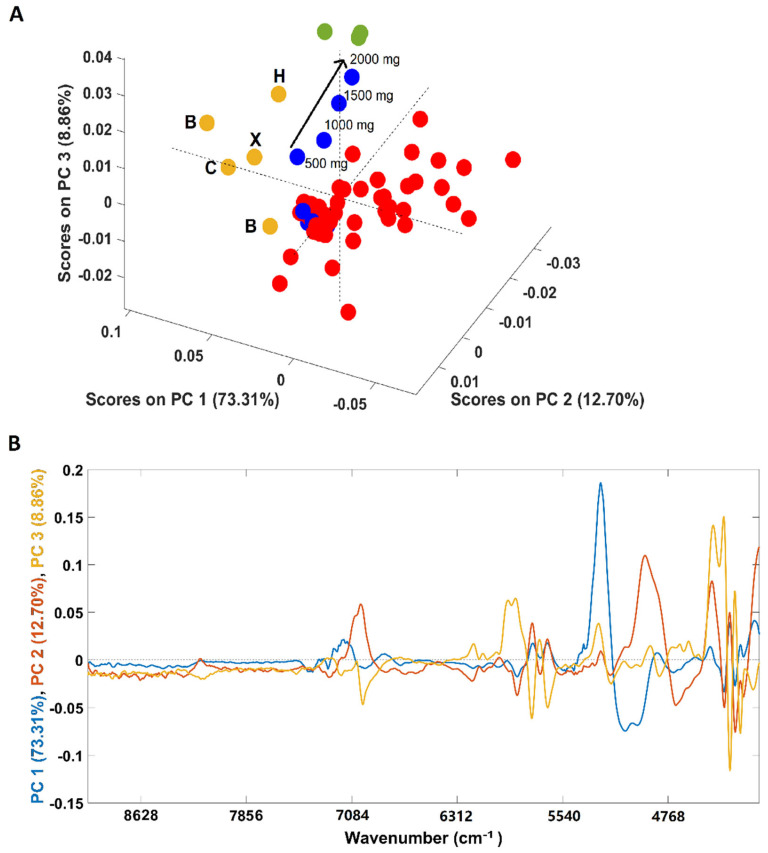
Scores plot of the PCA models developed solely with samples containing coffee (**A**) and its corresponding loadings (**B**). Legend: ● arabica (B = Brazil, H = Honduras, C = Colombia, X = blend of the 4 arabica samples); ● robusta; ● adulterated samples with robusta; ● adulterated samples with rice/corn/soy/barley/coffee husks.

An additional PCA was performed solely with the spectra of coffee samples (arabica, robusta and arabica adulterated with robusta) due to its closeness in the first PCA (Figure 2A). Both pure “arabica” and pure “robusta” coffee samples are clearly discriminated from the adulterated samples (all adulterated samples were included in the analysis) in the first PC (PC1). According to the loadings plot (Figure 2B), the spectral region responsible for the discrimination was 5150–4920 cm^−1^, a spectral region indicating the predominance of carbohydrates, proteins and chlorogenic acid vibrations in coffee samples [36]. Regarding the samples adulterated with “robusta” coffee, 4/8 samples were placed apart from the main cluster. These samples correspond to those with a higher “robusta” proportion (20/40/60/80%). Another interesting point is that the samples are positioned in the scores map according to their “robusta” proportion, e = with the sample with a higher content being closer to the pure “robusta” samples. Samples with lower “robusta” contents cluster together with the remaining adulterated samples. Regarding plain “arabica” samples (B1/B2/C/H and their blend X), it could be seen that samples from Brazil (B) and Colombia (C) are closer, lying mostly in the negative part of PC 3, while the sample from Honduras (H) is on the positive part of the PC 3. The loadings plot (Figure 1, panel IIB) shows that the regions between 5800 and 5650 cm^−1^ (vibration due to S-H and C-H bonds in first overtone) and between 4460 and 4270 cm^−1^ (dominated by carbohydrates, proteins and caffeine vibrations) are mainly responsible for the discrimination [36]. The green coffee processing method cannot be used to justify this separation since the Brazilian samples were processed by the natural method while the samples from Colombia and Honduras are washed coffees. Therefore, the relative location of the samples in the scores map could be related to their geographic origins. Colombia and Brazil are in South America, probably sharing many edaphoclimatic conditions, and Honduras is located in Central America. The geographic origin could justify the slightly different chemical composition suggested by the PCA. Previous studies on green coffee demonstrated the suitability of NIR spectroscopy to discriminate samples according to their geographical regions, while this work highlights a possible difference between roasted and ground coffees in terms of countries bases [37,38,39]. Precisely, following the findings of Giraudo and collaborators [40], the green samples from Honduras and Brazil showed a tendency towards separation. Since the “arabica” X sample corresponds to a balanced mix of all the four samples (B/B/C/H, 25% each) it is located closer to samples B and C due to their relative compositions (75% of B plus C and 25% of H).

### 3.2. Discrimination According to the Adulterant

**Figure 3:**
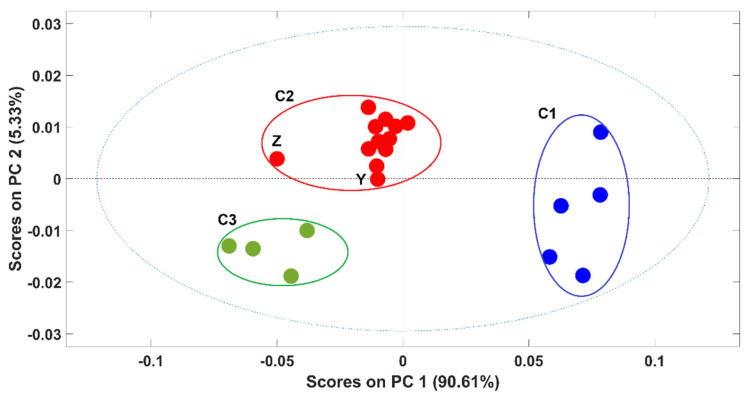
Scores plot of the first two principal components (PCs) of the PCA model. Legend: ● arabica; ● ≤10% of adulterants; ● >10% of adulterants. Samples Z and Y contain 10% of adulterants (5% of rice + 5% of coffee husks and 10% of rice, respectively).

Due to the high ability to discriminate between pure and contaminated samples, the potential of NIR spectroscopy to discriminate between samples according to the adulterants present was also evaluated. A PCA model was developed with spectra of pure arabica and arabica samples adulterated with rice (rice alone + all the adulterations with rice, alone and in combination with other adulterants). Figure 3 exhibits the scores plot of the first two PCs of the PCA model. The first PC (PC1), which captures 90.6% of the spectral variability, was responsible for the clear discrimination between arabica samples (cluster C1) and the contaminated ones (cluster C2 and C3) even in the presence of coffee husks and “robusta” coffee. The discrimination of these two clusters (C2 and C3) was related to the percentage of the adulterant present in the coffee sample and not with the kind of adulterant. Namely, samples with more than 10% of adulterants were in C3 and samples with less than 10% of adulterants were in C2, these last ones being closer to the arabica pure samples on the scores map of the PCA model. Included in C2 were only two samples’ spectra, containing exactly 10% of adulterants, one corresponds to spectra “Z”, with 5% of rice and 5% of coffee husks, and the second one with 10% of rice as the unique adulterant “Y”. The spectrum from sample Z was quite apart from the remaining ones probably due to the presence of coffee husks in a high percentage. It should be noted that despite containing 10% of adulterant, sample Y contains only rice as the adulterant, which makes this sample more similar to the others present in C2 (where all the samples containing only alteration with rice appeared). Similar PCA models were developed for each of the remaining adulterants and the obtained results were quite similar (data not shown).

Globally, it arises that sample discrimination according to the adulterant present was not possible. Instead, the discrimination observed in the scores map seems to be highly related to the total percentage of adulterants in the samples.

**Figure 4:**
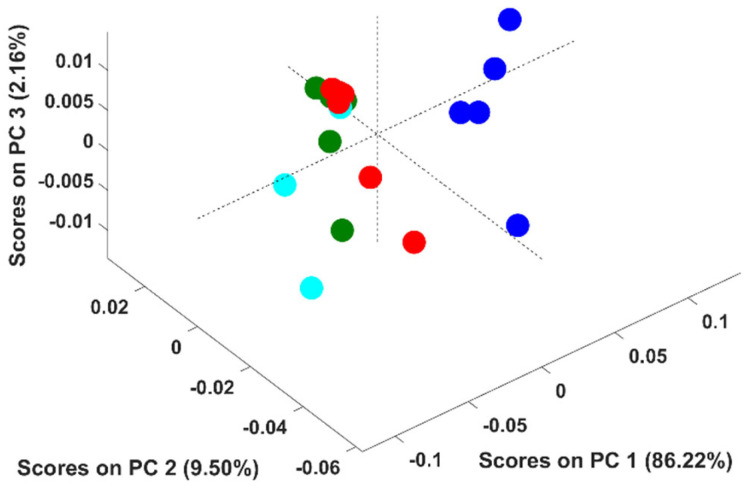
Scores plot of the first three principal components (PCs) of the PCA model. Legend: ● arabica; ● samples adulterated with coffee husks; ● samples adulterated with rice; ● samples adulterated with rice and coffee husks.

It should be stressed that the above conclusion was based on PCA models developed with adulterated samples with up to six adulterants simultaneously. In this context, an additional study was undertaken to evaluate if the discrimination according to the adulterant was feasible when solely up to two adulterants were present. Fifteen PCA models were developed (C 6,2- combinations of six adulterants, two by two) to include all the combinations. Figure 4 corresponds to the PCA model developed with adulterated samples containing rice and coffee husks for example proposes. Pure arabica samples were discriminated from the adulterated ones across the PC1 (86.2% of the spectral variability), as stated previously. Regarding the adulterated samples, some appeared in the scores map in a very compact cluster and others quite disperse across it. Samples belonging to the compact cluster possess percentages of coffee between 95 and 99.75%, which makes them all very similar even if they were adulterated with rice; coffee husks or rice + coffee husks. The dispersed ones possessed percentages of coffee ≤90% enabling the discrimination according to the adulterant present (rice/coffee husks/rice + coffee husks).

Similar results were obtained for the remaining PCA models developed (data not shown), meaning that the discrimination according to the adulterant present in the sample is only possible for percentages of adulterants ≥10% and with up to two adulterants. This result differs from the obtained previously because in the first attempt to discriminate samples according to the adulterant, some samples had very small amounts of 4 to 5 distinct adulterants.

### 3.3. Discrimination at a Constant Adulterant Concentration

**Figure 5:**
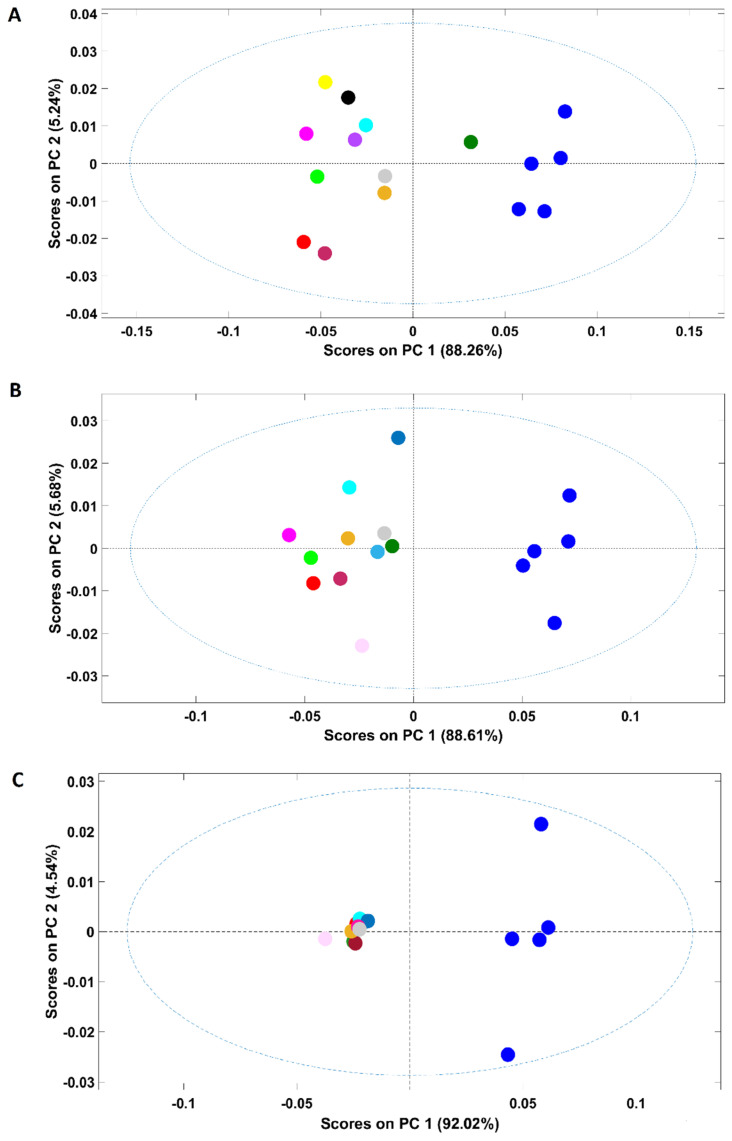
Scores plot of the PCA model developed with samples containing distinct percentages of adulterants: (**A**)—20%; (**B**)—10%; (**C**)—1%. Legend: ● pure arabica; ● robusta; ● corn; ● coffee husks; ● rice; ● soy; ● barley; ● coffee husks + barley; ● coffee husks + corn; ● coffee husks + rice; ● coffee husks + robusta; ● soy + coffee husks; ● barley + corn + soy + rice; ● barley + corn + soy + coffee husks; ● barley + corn + soy + robusta.

Based on the previous approaches, samples discrimination according to the adulterant might be possible if only up to two adulterants are considered. However, even in such conditions, the discrimination ability was highly related to the adulterant concentration (only feasible for adulterant concentration ≥10%). In this context, an additional study was performed to evaluate the feasibility of the discrimination according to the adulterant present keeping their concentration constant. Three PCA models were developed, each including solely samples of a certain adulterant concentration, namely, 20%, 10% and 1%. These percentages were selected based on the available data in order to ensure a representative range of adulterant amounts and based on the number of available spectra for each amount to develop robust PCA models. The scores plot of the PCA model developed with samples containing 20% of adulterant (Figure 5A) showed discrimination between samples containing just coffee (arabica and arabica adulterated with robusta) from adulterated coffee in the first PC (PC1 encompassing 88.3% of the spectral variability). Despite lying in the positive part of the PC1, plain arabica samples were discriminated from those adulterated with robusta. Adulterated samples with coffee husks and/or corn appear mostly on the negative part of PC2 while samples containing a mixture of adulterants and rice or soy plus coffee husks appear on the positive part of PC2. Even with a constant and quite high adulterant percentage in samples, when many adulterants were included, it seems to be not possible to discriminate samples according to the adulterants present.

Regarding samples with 10% of adulterant (scores map of the model in Figure 5B), a clear discrimination between pure arabica samples and adulterated ones occurred on PC1. Contrary to samples with 20% of adulterant, the arabica sample adulterated with robusta is on the opposite part (negative) of PC1. This might have occurred due to the lower percentage of adulterants in these samples, which make them more similar (all of them possess a higher arabica content, 90% versus 80% in the first case). The discrimination between arabica and robusta coffees is important, particularly for products labelled as 100% arabica. Adulterations with robusta are frequent due to its lower price (<20–25%), and it is frequently used to reduce the costs of the product [8,30]. Figure 5C presents the scores map of the PCA model developed with samples containing just 1% of adulterant. It was interesting to note that NIR spectroscopy possessed the ability to discriminate between pure and adulterated arabica samples even with a low percentage of adulterant (1%) on the first PC. Winkler-Moser et al. [7], in a single approach for corn detection in coffee using NIR, showed that the model developed using partial least-squares regression (PSLR) analysis was not able to detect samples at the 1% level, but an accurate detection by NIR was possible at or above 5%. The detection of corn in coffee was also effective by micro NIR (the limits of detection, LOD, and of quantification, LOQ, were 1.6 and 5.2%, respectively) [29]. In an additional work, barley adulteration was detected at 2% in coffee using PLSR [24]. It is important to highlight that the legislation in Brazil that allowed up to 1% of foreign material in roasted ground coffee through Normative Instruction nº 16 [41] was revoked by Normative Instruction nº 7 [42]. The results obtained in this work, allowing discrimination of adulteration below 1% of contribute to imposing the strict regulation of coffee products due to their high commercial value. Additionally, all of the adulterated samples appear in a very compact cluster, highlighting their similarity.

## 4. Conclusions

NIR spectroscopy coupled with chemometrics proved to be able to distinguish all the pure samples included in this work (coffee, including the two species arabica and robusta, coffee husks, barley, soybean, rice and corn).

This technique was also able to discriminate the coffee varieties among each other, namely, arabica, robusta and arabica contaminated with robusta from as low as 1%. Indeed, contaminated samples appeared positioned in the scores map according to their relative percentages. Additionally, pure arabica samples seem to be discriminated from each other according to their geographic origins.

The discrimination between pure and adulterated arabica coffee samples was also feasible for all the adulterants and independently of the concentration tested (from as low as 0.25%). However, the discrimination of the samples according to the adulterant present was only achievable if no more than two contaminants were present simultaneously and for adulterant concentrations ≥10%.

## Figures and Tables

**Table 1:** List of the coffee samples and adulterants according to origin and degree of roasting.

Sample	Origin	Roasting Condition
Arabica B1	Brazil (natural)	medium-dark
Arabica B2	Brazil (natural)	medium-dark
Arabica C	Colombia (washed)	medium-dark
Arabica H	Honduras (washed)	medium-dark
Robusta 1	Vietnam	medium-dark
Robusta 2	Cameroon	medium-dark
Corn 1	Brazil	225 °C 30 min
Corn 2	Portugal	250 °C 45 min
Soybeans 1	Portugal	250 °C 15 min
Soybeans 2	Portugal	250 °C 15 min
Rice seeds (with chaff) 1	Brazil	250 °C 25 min
Rice seeds (with chaff) 2	Portugal	250 °C 30 min
Coffee husks 1	Brazil	220 °C 10 min
Coffee husks 2	Brazil	212 °C 14 min
Barley 1	Portugal	commercial
Barley 2	Portugal	commercial

## Data Availability

No new data were created or analyzed in this study. Data sharing is not applicable to this article.

## Supplementary Materials

The following are available online at https://www.mdpi.com/article/10.3390/foods11010061/s1, Table S1: blends composition, Table S2. Prevalence of each adulterant in the blends, Table S3. Root mean square errors of calibration (RMSEC) and cross-validation (RMSECV) of the PCA models developed in this study. PCA models were identified through their figure numbers in the manuscript.
