# Supplementary material for: Near-Infrared Spectroscopy Applied to the Detection of Multiple Adulterants in Roasted and Ground Arabica Coffee
Source: Foods. 2021 Dec 28;11(1):61. doi: 10.3390/foods11010061 (PMC8750839; doi:10.3390/foods11010061)
Supplement: Supplementary file 1 [file foods-11-00061-s001.zip › foods-1489565-supplementary.pdf]

Table S1. Blends composition.

Blend (%)			
**Natural Arabica Coffee Brazil**	**Natural Arabica Coffee Brazil**	**Washed Arabica Coffee Colombia**	**Washed Arabica Coffee Honduras**
B1			
	B2		
		C	
			H
Arabica X			

**Barley 1**	**Barley 2**	**Rice 1**	**Rice 2**
Barley Y		Rice Y	

**Corn 1**	**Corn 2**	**Coffee husks 1**	**Coffee husks 2**
Corn Y		Coffee husks Y	

**Soy 1**	**Soy 2**	**Robusta 1**	**Robusta 2**
Soy Y		Robusta Y	

**Arabica X**	**Barley Y**	**Corn Y**	**Soy Y**	**Rice Y**	**Coffee husks Y**	**Robusta Y**
99.8	0.25	-	-	-	-	-
99.8	-	0.25	-	-	-	-
99.8	-	-	0.25	-	-	-
99.8	-	-	-	0.25	-	-
99.8	-	-	-	-	0.25	
99.8	-	-	-	-	-	0.25
99.5	0.5	-	-	-	-	-
99.5	-	0.5	-	-	-	-
99.5	-	-	0.5	-	-	-
99.5	-	-	-	0.5	-	-
99.5	-	-	-	-	0.5	-
99.5	-	-	-	-	-	0.5
99.0	0.5	-	-	-	0.5	-
99.0	-	0.5	-	-	0.5	-
99.0	-	-	0.5	-	0.5	-
99.0	-	-	-	0.5	0.5	-
99.0	-	-	-	-	0.5	0.5
98.5	0.5	0.5	0.5	-	-	-
98.5	-	0.5	-	-	0.5	0.5
98.0	0.5	0.5	0.5	-	-	0.5
98.0	0.5	0.5	0.5	0.5	-	-
98.0	0.5	0.5	0.5	-	0.5	-
97.5	0.5	0.5	0.5	0.5	0.5	-
99.0	1	-	-	-	-	-
99.0	-	1	-	-	-	-
99.0	-	-	1	-	-	-
99.0	-	-	-	1	-	-
99.0	-	-	-	-	1	-
99.0	-	-	-	-	-	1
98.0	1	-	-	-	1	-
98.0	-	1	-	-	1	-
98.0	-	-	1	-	1	-

98.0	-	-	-	1	1	-
98.0	-	-	-	-	1	1
97.0	1	1	1	-	-	-
97.0	-	1	-	-	1	1
96.0	1	1	1	1	-	-
96.0	1	1	1	-	1	
96.0	1	1	1	-	-	1
95.0	1	1	1	1	1	-
95.0	5	-	-	-	-	-
95.0	-	5	-	-	-	-
95.0	-	-	5	-	-	-
95.0	-	-	-	5	-	-
95.0	-	-	-	-	5	-
95.0	-	-	-	-	-	5
90.0	5	-	-	-	5	-
90.0	-	5	-	-	5	-
90.0	-	-	5	-	5	-
90.0	-	-	-	5	5	-
90.0	-	-	-	-	5	5
85.0	5	5	5	-	-	
85.0	-	5	-	-	5	5
80.0	5	5	5	5	-	
80.0	5	5	5	-	-	5
80.0	5	5	5	-	5	-
75.0	5	5	5	5	5	-
70.0	5	5	5	5	5	5
90.0	10	-	-	-	-	-
90.0	-	10	-	-	-	-
90.0	-	-	10	-	-	-
90.0	-	-	-	10	-	-
90.0	-	-	-	-	10	-
90.0	-	-	-	-	-	10
80.0	10	-	-	-	10	-
80.0	-	10	-	-	10	-
80.0	-	-	10	-	10	-
80.0	-	-	-	10	10	-
80.0	-	-	-	-	10	10
80.0	-	20.0	-	-	-	-
80.0	-	-	-	-	20.0	-
80.0	-	-	-	-	-	20.0
60.0	-	-	-	-	-	40.0
40.0	-	-	-	-	-	60.0
20.0	-	-	-	-	-	80.0

All samples were prepared in triplicate.
X- 25% mixture of B1:B2:C:H; Y- 50% mixture of the adulterant batch 1 and 2.

Table S2. Prevalence of each adulterant in the blends

%	Barley	Corn	Soybean	Rice	coffee husks	Robusta coffee
	number of combinations prepared with each of the adulterants					
0,25	1	1	1	1	1	-
0,5	7	7	7	4	9	4
1	7	7	7	4	9	4
5	7	7	7	4	9	4
10	2	2	2	2	6	2
20	-	1	-	-	1	1
40	-	-	-	-	-	1
60	-	-	-	-	-	1
80	-	-	-	-	-	1

Table S3. Root mean square errors of calibration (RMSEC) and cross-validation (RMSECV) of the PCA models developed in this study. PCA models were identified through their figure numbers in the manuscript.

PCA model	RMSEC	RMSECV
**Figure 1**	3.8×10^{-5}	1.8×10^{-4}
**Figure 2**	9.1×10^{-5}	1.4×10^{-4}
**Figure 3**	1.1×10^{-4}	2.5×10^{-4}
**Figure 4**	1.7×10^{-4}	3.0×10^{-4}
**Figure 5A**	1.6×10^{-4}	3.5×10^{-4}
**Figure 5B**	1.8×10^{-5}	3.1×10^{-4}
**Figure 5C**	8.2×10^{-5}	3.3×10^{-4}
